# Improved Geometric Path Enumeration for Verifying ReLU Neural Networks

**DOI:** 10.1007/978-3-030-53288-8_4

**Published:** 2020-06-13

**Authors:** Stanley Bak, Hoang-Dung Tran, Kerianne Hobbs, Taylor T. Johnson

**Affiliations:** 8 Microsoft Research Lab, Redmond, WA USA; 9 University of Southern California, Los Angeles, CA USA; 10 Stony Brook University, Stony Brook, USA; 11 University of Nebraska, Lincoln, USA; 12 Vanderbilt University, Nashville, USA; 13 Air Force Research Laboratory, Wright-Patterson Air Force Base, USA; 14 Georgia Institute of Technology, Atlanta, USA

## Abstract

Neural networks provide quick approximations to complex functions, and have been increasingly used in perception as well as control tasks. For use in mission-critical and safety-critical applications, however, it is important to be able to analyze what a neural network can and cannot do. For feed-forward neural networks with ReLU activation functions, although exact analysis is NP-complete, recently-proposed verification methods can sometimes succeed.

The main practical problem with neural network verification is excessive analysis runtime. Even on small networks, tools that are theoretically complete can sometimes run for days without producing a result. In this paper, we work to address the runtime problem by improving upon a recently-proposed geometric path enumeration method. Through a series of optimizations, several of which are new algorithmic improvements, we demonstrate significant speed improvement of exact analysis on the well-studied ACAS Xu benchmarks, sometimes hundreds of times faster than the original implementation. On more difficult benchmark instances, our optimized approach is often the fastest, even outperforming inexact methods that leverage overapproximation and refinement.

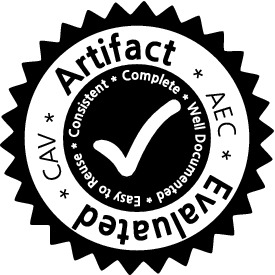



## Introduction

Neural networks have surged in popularity due to their ability to learn complex function approximations from data. This ability has led to their proposed application in perception and control decision systems, which are sometimes safety-critical. For use in safety-critical applications, it is important to prove properties about neural networks rather than treating them as black-box components.

A recent method [24] based on path enumeration and geometric set propagation has shown that exact analysis can be practical for piecewise linear neural networks. This includes networks with fully-connected layers, convolutional layers, average and max pooling layers, and neurons with ReLU activation functions. Here, we focus on fully-connected layers with ReLU activation functions. The verification problem in this method is presented in terms of input/output properties of the neural network. The method works by taking the input set of states and performing a set-based execution of the neural network. Due to the linear nature of the set representation and the piecewise linear nature of the ReLU activation function, the set may need to be split after each neuron is executed, so that the output after the final layer is a collection of sets that can each be checked for intersection with an unsafe set.

Since the formal verification problem we are addressing has been shown to be NP-Complete [13], we instead focus on improving practical scalability. This requires us to choose a set of benchmarks for evaluation. For this, we focus on properties from the well-studied ACAS Xu system [13]. This contains a mix of safe and unsafe instances, where the original verification times measured from seconds to days, including some unsolved instances.

The main contributions of this paper are:

- several new speed improvements to the path enumeration method, along with correctness justifications, that are each systematically evaluated;
- the first verification method that verifies all 180 benchmark instances from ACAS Xu properties 1–4, each in under 10 min on a standard laptop;
- a comparison with other recent tools, including Marabou, Neurify, NNV, and ERAN, where our method is often the fastest and over 100x faster than the original path enumeration method implementation in NNV.

This paper first reviews background related to neural networks, the path enumeration verification approach, and the ACAS Xu benchmarks in Sect. 2. Next, Sect. 3 analyzes several algorithmic optimizations to the basic procedure, and systematically evaluates each optimization’s effect on the execution times of the ACAS Xu benchmarks. A comparison with other tools is provided in Sect. 4, followed by review of related work in Sect. 5 and a conclusion.

## Background

We now review the neural network verification problem (Sect. 2.1), the basic geometric path enumeration algorithm (Sect. 2.2), important spatial data structures (Sect. 2.3), and the ACAS Xu benchmarks (Sect. 2.4).

### Neural Networks and Verification

In this work, we focus our attention on fully-connected, feedforward neural networks with ReLU activation functions. A neural network computes a function $\textsf {NN}: \mathbb {R}^{n_i} \rightarrow \mathbb {R}^{n_o}$, where $n_i$ is the number of inputs and $n_o$ is the number of outputs. A neural network consists of *k* layers, where each layer *i* is defined with a weight matrix $W_i$ and a bias vector $b_i$. Given an input point $y_0 \in \mathbb {R}^{n_i}$, a neural network will compute an output point $y_k \in \mathbb {R}^{n_o}$ as follows:We call $y_{i-1}$ and $y_i$ the input and output of the *i*-th layer, respectively, and $x^{(i)}$ the intermediate values at layer *i*. The vector-function *f* is defined using a so-called *activation function*, that is applied element-wise to the vector of intermediate values at each layer. We focus on the popular rectified linear unit (ReLU) activation function, $\textsf {ReLU}(x) = \max (x, 0)$.

For this computation definition to make sense, the sizes of the weights matrices and bias vectors are restricted. The first layer must accept $n_i$-dimensional inputs, the final layer must produce $n_o$-dimensional outputs, and the intermediate layers must have weights and biases that have sizes compatible with their immediate neighbors, in the sense of matrix/vector multiplication and addition. The number of neurons (sometimes called hidden units) at layer *i* is defined as the number of elements in the layer’s output vector $y_i$.

#### Definition 1 (Output Range)

Given a neural network that computes the function NN and an input set $\mathcal {I} \subseteq \mathbb {R}^{n_i}$, the **output range** is the set of possible outputs of the network, when executed from a point inside the input set, .

Computing the output range is one way to solve the verification problem.

#### Definition 2 (Verification Problem for Neural Networks)

Given a neural network that computes the function NN, an input set $\mathcal {I} \subseteq \mathbb {R}^{n_i}$, and an unsafe set $\mathcal {U} \subseteq \mathbb {R}^{n_o}$, the **verification problem for neural networks** is to check if .

If verification is impossible, we would also prefer to generate a counterexample $y_0 \in \mathcal {I}$ where $y_k = \textsf {NN}(y_0)$ and $y_k \in \mathcal {U}$, although not all tools do this. We also further assume in this work that the input and unsafe sets are defined with linear constraints, , and .

### Basic Geometric Path Enumeration Algorithm

Given enough time, the output range of a neural network can be computed exactly using a recently-proposed geometric path enumeration approach [24]. The general strategy is to execute the neural network with *sets* instead of points. A *spatial data structure* is used to represent the input set of states, and this set is propagated through each layer of the neural network, computing the set of possible intermediate values and then the set of possible outputs repeatedly until the output of the final layer is computed. In this context, a spatial data structure represents some subset of states in a Euclidean space $\mathbb {R}^n$, where the number of dimensions *n* is the number of neurons in one of the layers of the network, and may change as the set is propagated layer by layer. An example spatial data structure could be a polytope defined using a finite set of half-spaces (linear constraints), although as explained later this is not the most efficient choice. Section 2.3 will discuss spatial data structures in more detail.

The high-level verification method is shown in Algorithm 1, where functions in red are custom to the spatial data structure being used. The  function (line 1) converts the input set $\mathcal {I}$ from linear constraints to the desired spatial data structure, and stores it in the $\theta$ element of *s*, where *s* is called a *computation-state tuple*. A neuron value of None in the tuple indicates that next operation should be an affine transformation. The computation-state tuple is then put into a waiting list (line 3), which stores tuples that need further processing. The step function (line 7) propagates the set $\theta$ by a single neuron in a single layer of the network, and is elaborated on in the next paragraph. This function can modify $\mathcal {W}$, possibly inserting one or more computation-state tuples, although always at a point further along in the network (with a larger layer number or neuron index), which ensures eventual termination of the loop. This function will also check if the set, after being fully propagated through the network, intersects the unsafe set. In this case, step will return unsafe, which causes the while loop to immediately terminate since the result is known.
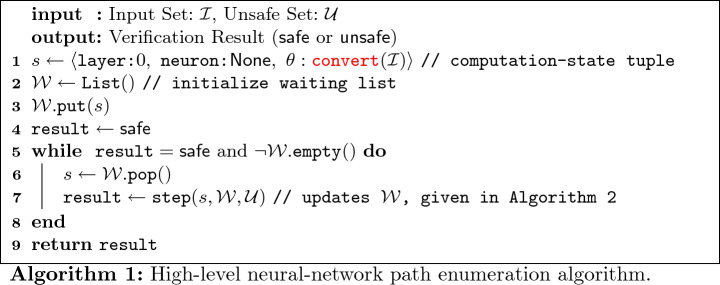
 
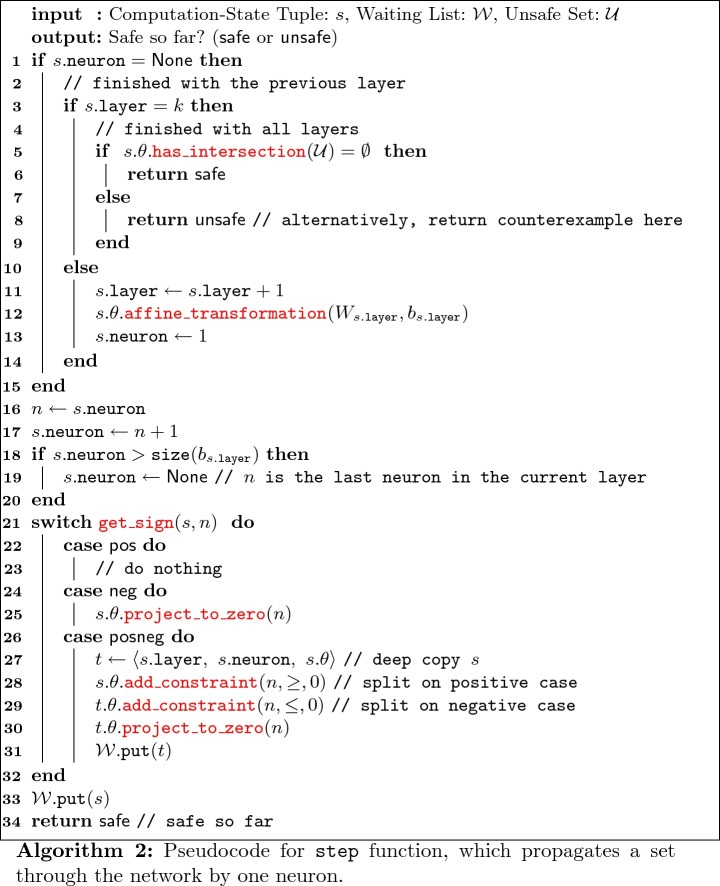


The step function propagates the set of states $\theta$ by one neuron, and is shown in Algorithm 2. The intermediate values are computed from the input set of each layer by calling  (line 12). For the current neuron index *n*, the algorithm will check if the input to the ReLU activation function, dimension *n* of the set $\theta$, is always positive (or zero), always negative, or can be either positive or negative. This is done by the  function (line 21), which returns pos, neg, or posneg, respectively. In the first two cases, the current dimension *n* of the set is left alone or assigned to zero (using the  method), to reflect the semantics of the ReLU activation function when the input is positive or negative, respectively. In the third case, the set is split into two sets along linear constraint where the input to the activation function equals zero. In the case where the input to the activation function is less than zero, the value of dimension *n* is projected to zero, reflecting the semantics of the ReLU activation function. The splitting is done using the  method of the spatial data structure, which takes three arguments: *n*, sign, and val. This method intersects the set with the linear condition that the *n*-th dimension is, depending on sign, greater than, less than, and/or equal to val. Once the set has been propagated through the whole network, it is checked for intersection with the unsafe set (line 5), using the  method.

This enumeration algorithm has been shown to be sound and complete [24]. However, for this strategy to work in practice, the spatial data structure used to store $\theta$ must support certain operations *efficiently*. These are denoted in red in Algorithms 1 and 2:  ,  ,  ,  ,  , and  . Polytopes represented with half-spaces, for example, do not have a known efficient way to compute general affine transformations in high dimensions. Instead, linear star sets [4] will be used, which are a spatial data structure that support all the required operations efficiently and without overapproximation error. These will be elaborated on more in the next subsection.

In this work, we focus on optimizations to the presented algorithm that increase its practical scalability, while exploring the same set of paths. The most important factor that we do not control and influences whether this can succeed is the number of paths that exist. Each output set that gets checked for intersection with the unsafe set corresponds to a unique *path* through the network, where the path is defined by the sign of each element of the intermediate values vector at each layer. The algorithm enumerates every path of the network for a given input set. An upper bound on this is $2^N$, where *N* is the total number of neurons in all the layers of the network. For many practical verification problem instances, however, the actual number of unique paths is significantly smaller than the upper bound.

### Spatial Data Structures

Using the correct spatial data structure (set representation in this context) is important to the efficiency of Algorithm 1 and 2, as well as some of our optimizations. Here we review two important spatial data structures, zonotopes and (linear) star sets.

**Zonotopes.** A *zonotope* is an affine transformation of the $[-1, 1]^p$ box. Zonotopes have been used for efficient analysis of hybrid systems [8] as well as more recently to verify neural networks using overapproximations [7, 21]. Zonotopes can be described mathematically as $Z = (c, G)$, where the *center* *c* is an *n*-dimensional vector and *generator matrix* *G* is an $n \times p$ matrix. The columns of *G* are sometimes referred to as *generators* of the zonotope, and we write these as $g_1, \ldots , g_p$. A zonotope *Z* encodes a set of states as:1The two most important properties of zonotopes for the purposes of verification are that they are efficient for (i) affine transformation, and (ii) optimization.

An affine transformation of an *n*-dimensional point *x* to a *q*-dimensional space is defined with a $q \times n$ matrix *A* and *q*-dimensional vector *b* so that the transformed point is $x' = Ax + b$. An affine transformation of every point in an *n*-dimensional set of points described by a zonotope $Z = (c, G)$ is easily computed as $Z' = (Ac + b, AG)$. Note this uses standard matrix operations which scale polynomially with the dimension of *A*, and are especially efficient if the number of generators is small. In the verification problem, the number of generators, *p*, corresponds to the degrees of freedom needed to encode the input set of states. In ACAS Xu system, for example, there are 5 inputs, and so the input set can be encoded with 5 generators. In contrast, affine transformations of polytopes require converting between a half-space and vertex representation, which is slow.

The second efficient operation for zonotopes is optimization in some direction vector *v*. Given a zonotope $Z = (c, G)$ and a direction *v* to maximize, the point $x^* \in Z$ that maximizes the dot product $v \cdot x^*$ can be obtained as a simple summation $x^* = c + \sum _{i=1}^p x_i^*$, where each $x_i^*$ is given as:

2 $$\begin{aligned} x_i^* = {\left\{ \begin{array}{ll} v_i, &{} \text {if } v_i \cdot g_i \ge - v_i \cdot g_i \\ -v_i, &{} \text {otherwise} \end{array}\right. } \end{aligned}$$

**Star Sets.** A (linear) *star set* is another spatial data structure that generalizes a zonotope. A star set is an affine transformation of an arbitrary *p*-dimensional polytope. Mathematically, a star set *S* is a 3-tuple, (*c*, *G*, *P*), where *c* and *G* are the same as with a zonotope, and *P* is a half-space polytope in *p* dimensions. A star set *S* encodes a set of states (compare with Eq. 1):3A star set can encode any zonotope by letting *P* be the $[-1, 1]^p$ box. Star sets can also encode more general sets than zonotopes by using a more complex polytope *P*. A triangle, for example, can be encoded as a star set by setting *P* to be a triangle, using the origin as *c* and the identity matrix as *V*. This cannot be encoded with zonotopes, as they must be centrally symmetric. In Algorithm 1 on line 1, the  function produces the input star set (*c*, *G*, *P*) from input polytope $\mathcal {I}$ setting *c* to the zero vector, *G* to the identity matrix, and *P* to $\mathcal {I}$.

Affine transformations by a $q \times n$ matrix *A* and *q*-dimensional vector *b* of a star set *S* can be computed efficiently similar to a zonotope: $S' = (Ac + b, AG, P)$.

Optimization in some direction *v* is slightly less efficient than with a zonotope, and can be done using linear programming (LP). To find a point $x^* \in S$ that maximizes the dot product $v \cdot x^*$, we convert the optimization direction *v* to the initial space $w = (vG)^T$, find a point $\alpha ^* \in P$ that maximizes *w* using LP, and then convert $\alpha ^*$ back to the *n*-dimensional space $x^* = c + G \alpha ^*$.

Star sets, unlike zonotopes, also efficiently support half-space intersection operations by adding constraints to the star set’s polytope. Given a star set $S = (c, G, P)$ and an *n*-dimensional half-space $d x \le \texttt {e}$ defined by vector *d* and scalar $\texttt {e}$, we convert this to a *p*-dimensional half-space as follows:

4 $$\begin{aligned} (d G) \alpha \le \texttt {e} - dc \end{aligned}$$

The star set after intersection is then $S' = (c, G, P')$, where the half-space polytope $P'$ is the same as *P*, with one additional constraint given by Eq. 4.

### ACAS Xu Benchmarks

Since the verification problem for neural networks is NP-Complete, we know exact analysis methods cannot work well in all instances. In order to evaluate improvements, therefore, we must focus on a set of benchmarks.

In this work, we choose to focus on the Airborne Collision System X Unmanned (ACAS Xu) set of neural network verification benchmarks [13]. As these benchmarks have been widely-used for evaluation in other publications, and some authors have even made their tools available publicly, using these allows us to provide a common comparison point with other methods later in Sect. 4.

ACAS Xu is a flight-tested aircraft system designed to avoid midair collisions of unmanned aircraft by issuing horizontal maneuver advisories [17]. The system was designed using a partially observable Markov decision process that resulted in a 2 GB lookup table which mapped states to commands. This mapping was compressed to 3 MB using 45 neural networks (two of the inputs were discretized and are used to choose the applicable network) [12]. Since the compression is not exact, the verification step checks if the system still functions correctly.

Each network contains five inputs that get set to the current the aircraft state, and five outputs that determine the current advisory. The network has six ReLU layers with 50 neurons each, for a total of 300 neurons. Ten properties were originally defined, encoding things like, if the aircraft are approaching each other head-on, a turn command will be advised (property 3). The formal definition of all the properties encoded as linear constraints is available in the appendix of the original work [13].

## Improvements

We now systematically explore several improvements to the exact path enumeration verification method from Sect. 2.2. For each proposed improvement, we compare the run-time on the ACAS Xu system with and without the change. We focus on properties 1–4. Although originally these were measured on a subset of the 45 networks [13], the same authors later used all the networks to check these properties [14], which is what we will do here. Each verification instance is run with a 10 min timeout, so that the maximum time needed to test a single method, if a timeout is encountered on each of the 180 benchmarks, is 30 h. Later, in Sect. 4, we will compare the most optimized method with other verification tools and the other ACAS Xu properties. Unless indicated otherwise, our experiments were performed on a Laptop platform with Ubuntu Linux 18.04, 32 GB RAM and an Intel Xeon E-2176M CPU running at 2.7 GHz with 6 physical cores (12 virtual cores with hyperthreading). The full data measurements summarized in this section are provided in Appendix C.

### Local Search Type (DFS vs BFS)

Algorithm 1 uses a waiting list to store the computation-state tuples, which are popped off one at a time and passed to the step function. This need not strictly be a list, but is rather a collection of computation-state tuples, and we can consider changing the order states are popped to explore the state space with different strategies. If the possible paths through the neural network are viewed as a tree, two well-known strategies for tree traversal that can be considered are depth-first search (DFS) and breadth-first search (BFS). A DFS search can be performed popping the computation-state tuple with the largest $(\textsf {layer}, \textsf {neuron})$ pair, whereas a BFS search is done by popping the tuple with the smallest $(\textsf {layer}, \textsf {neuron})$ pair.

**Fig. 1.**
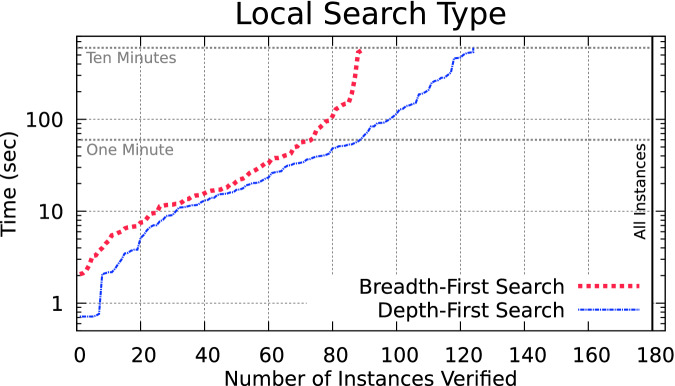
Depth-first search outperforms breadth-first search.

The original path enumeration with star set approach [24] describes a layer-by-layer exploration strategy, which is closer to a BFS search. Finite-state machine model-checking methods, however, more often use DFS search.

We compare the two approaches in Fig. 1, which summarizes the execution of all 180 benchmarks. Here, the *y*-axis is a timeout in seconds, and the *x*-axis is the number of benchmarks verified within that time. Within the ten minute timeout, around 90 benchmarks can be successfully verified with BFS, and 120 with DFS^1^. Notice that the *y*-axis is log scale, so that differences in runtimes between easy and hard benchmark instances are both visible.

As can be seen in the figure, the DFS strategy is superior. This is primarily due to unsafe instances of the benchmarks, where DFS can often quickly find an unsafe execution and exit the high-level loop, whereas BFS first iterates through all the layers and neurons (DFS explores deep paths, which sometimes are quickly found to be unsafe). In the cases where the system was safe, both approaches took similar time. Another known advantage of DFS search is that the memory needed to store the waiting list is significantly smaller, which can be a factor for the benchmarks with a large number of paths.

**Correctness Justification:** Both DFS and BFS explore the same sets of states, just in a different order.

### Bounds for Splitting

Using DFS search, we consider other improvements. The original path enumeration publication mentions the following optimization:

“$\ldots$ to minimize the number of [operations] and computation time, we first determine the ranges of all states in the input set which can be done efficiently by solving $\ldots$ linear programming problems.” [24]

An evaluation of the improvement is not provided, so we investigate this here. The optimization is referring to the implementation of the  function on line 21 of Algorithm 2. The  function takes as input a computation-state tuple *s* with spatial data structure $\theta$ (a star set) and a dimension number *n*. It returns pos, neg, or posneg, depending on whether value of dimension *n*, which we call $x_n$, in set $\theta$ can be positive (or zero), negative or both. Our baseline implementation, which we refer to as Copy, determines the output of  by creating two copies of the passed-in star set, intersecting them with the condition that $x_n \le 0$ or $x_n \ge 0$, and then checking each star set for feasibility, done using linear programming (LP). In the second version, which we call Bounds, the passed-in star set is instead minimized and maximized in the direction of $x_n$, to determine the possible signs. While Copy incurs overhead from creating copies and adding intersections, Bounds does extra work by computing the minimum and maximum which are not really needed (we only need the possible signs of $x_n$).

**Fig. 2.**
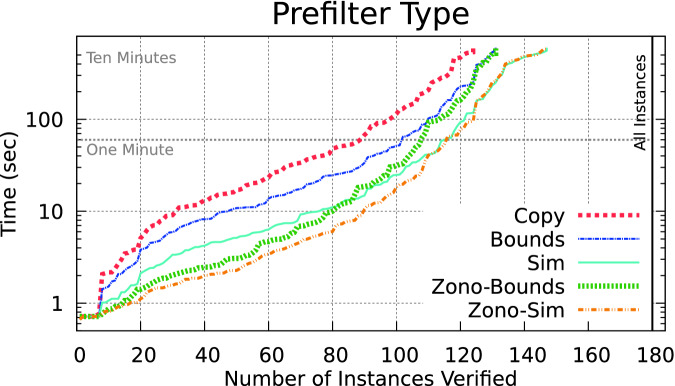
Prefilter optimizations improve performance by rejecting branches without LP solving. The Zono-Sim method works best.

A comparison of the optimizations on the ACAS Xu benchmarks are shown in Fig. 2 by comparing Copy to Bounds, we confirm the original paper’s claim that Bounds is faster.

**Correctness Justification:** If $\theta$ intersected with $x_n \le 0$ is feasible, then the minimum value of $x_n$ in $\theta$ will be less than or equal to zero and vice versa. Similar for the maximum case.

### Fewer LPs with Concrete Simulations

We next consider strategies to determine the possible signs of a neuron’s output with fewer LP calls, which we call *prefiltering*. Consider a modification of the Bounds optimization, where rather than computing both the upper and lower bound of $x_n$, we first compute the lower bound and check if its value is positive. If this is the case, we know  should return pos, and we do not need to compute the upper bound. We could, alternatively, first compute the upper bound and check if its value is negative. If there is no branching and we guess the correct side to check, only a single LP needs to be solved instead of two.

We can do even better than guessing by tracking extra information in the computation-state tuple. We add a simulation field to *s*, which contains a concrete value in the set of states $\theta$. This is initialized to any point in the input set $\mathcal {I}$, which can be obtained using LP, or using the center point if the input states are a box. When  returns posneg and the set is split (line 27 in Algorithm 2), the optimization point $x^*$ that proved a split was possible is used as the value of simulation in the new set. Also, when an affine transformation of the set is computed (line 12 in Algorithm 2), or when the set is projected to zero, simulation must also be modified by the same transformation.

With a concrete value of $x_n$ available in simulation, we use its sign to decide whether to first check the upper or lower bound of dimension *n* in $\theta$. If the *n*th element of simulation is positive, for example, we first compute the lower bound. If this is positive (or zero), then  can return pos. If the lower bound is negative, then we can immediately return posneg without solving another LP, since the simulation serves as a witness that $x_n$ can also be positive. Only when the simulation value of $x_n$ is zero do we need to solve two LPs.

We call this method Sim in Fig. 2. This is shown to be generally faster than the previous methods, as the overhead to track simulations is small compared with the gains of solving fewer LPs.

**Correctness Justification:** If the lower bound of $x_n$ is greater than zero, than its upper bound will be also be greater than zero and pos is the correct output. If the lower bound is less than zero and the *n*th element of simulation is greater than zero, than the upper bound will also be positive, since it must be greater than or equal to the value in the simulation (simulation is always a point in the set $\theta$), and so posneg is correct. Similar for the opposite case.

### Zonotope Prefilter

We can further reduce LP solving by using a zonotope. In each computation-state tuple *s*, we add a zonotope field *z* that overapproximates $\theta$, so that $\theta \subseteq z$. In the ACAS Xu benchmarks (and most current benchmarks for verification of NNs), the input set of states is provided as interval values on each input, which is a box and can be used to initialize the zonotope. Otherwise, LPs can be solved to compute box bounds on the input set to serve as an initial value. During the affine transformation of $\theta$ (line 12 in Algorithm 2), the zonotope also gets the same transformation applied. Cases where $\theta$ gets projected to zero are also affine transformations and can be exactly computed with the zonotope *z*. The only unsupported operation in the algorithm for zonotopes is  , used during the splitting operation (lines 28–29 in Algorithm 2). We skip these operations for the zonotope, which is why *z* is an overapproximation of $\theta$.

With a zonotope overapproximation *z* available during  , we can sometimes reduce the number of LPs to zero. Computing the minimum and maximum of the *n*-th dimension of *z* is an optimization problem over zonotopes, which recall from Sect. 2.3 can be done efficiently as a simple summation. If the *n*-th dimension of *z* is completely positive or negative, we can return pos or neg immediately. Otherwise, if both positive and negative values are possible in the zonotope, we fall back to LP solving on $\theta$ to compute the possible signs. This can be done either by computing both bounds, which we call Zono-Bounds or with the simulation optimization from before, which we call Zono-Sim. The performance of the methods are shown in Fig. 2. The Zonotope-Sim method performs the fastest, verifying about 145 benchmarks in under 10 min and demonstrating that reduction in LP solving is worth the extra bookkeeping.

**Correctness Justification:** Rejecting branches without LP solving is justified by the fact that *z* is an overapproximation of $\theta$. This is initially true, as if the input set is a box then $z = \theta$ and otherwise *z* is the box overapproximation of $\theta$. This is also true for every operation other than  , as these are exact for zonotopes. Finally, it is also true when  operation is skipped on *z*, as adding constraints can only reduce the size of the set $\theta$. If $\theta \subseteq z$, every smaller set $\theta '$ will also be a subset of *z* by transitivity, $\theta ' \subseteq \theta \subseteq z$, and so an overapproximation is maintained by ignoring these operations with *z*. Finally, if the *n*-th dimension of an overapproximation of $\theta$ is strictly positive (or negative), the *n*-th dimension of $\theta$ will also be strictly positive (or negative).

### Eager Bounds Computation

The step function shown in Algorithm 2 computes the sign of $x_n$ for the current neuron *n*. An alternative approach is to compute the possible signs for every neuron’s output in the current layer immediately after the affine transformation on line 12. These bounds can be saved in the computation-state tuple *s* and then accessed by  . The potential advantage is that, if a split is determined as impossible for some neuron *n*, and a split occurs at some earlier neuron $i < n$, then the split will also be impossible for neuron *n* in both of the sets resulting from the earlier split at neuron *i*. In this way, computing the bounds once for neuron *n* is sufficient in the parent set, as opposed to computing the bounds twice, in each of the two children sets resulting from the split. The benefit can be even more drastic if there are multiple splits before neuron *n* is processed, where potentially an exponential number of bounds computations can be skipped due to a single computation in the parent. On the other hand, if a split is possible, we will have computed more bounds than we needed, as we will do the computation once in the parent and then once again in each of the children. Furthermore, this method incurs additional storage overhead for the bounds, as well as copy-time overhead when computation-state tuples are deep copied on line 27. Experiments are important to check if the benefits outweigh the costs.

**Fig. 3.**
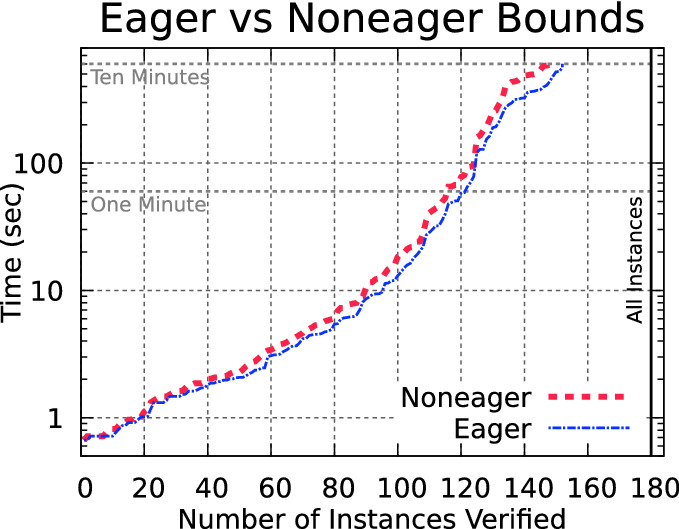
Computing neuron output bounds eagerly improves speed.

The modified algorithm, which we call Eager, will use the zonotope prefilter and simulation as before to compute the bounds, but this will be done immediately after the affine transformation on line 12. Further, when a split occurs along neuron *n* in the posneg case, the bounds also get recomputed in the two children for the remaining neurons in the layer, starting at the next neuron $n+1$. Neurons where a split was already rejected do not have their bounds recomputed. This algorithm is compared with the previous approach, called Noneager. In Fig. 3, we see eager computation of bounds slightly improves performance.

**Fig. 4.**
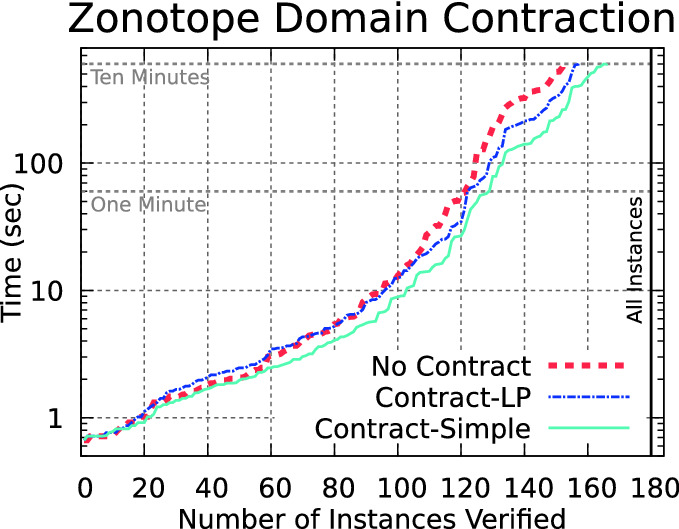
Zonotope domain contraction improves overall performance.

**Correctness Justification:** When sets are split in the posneg case in Algorithm 2, each child’s $\theta$ is a subset of the parent’s $\theta$. Thus, the upper and lower bound of the output of each neuron *n* can only move inward. Thus, if the parent’s bounds for some neuron are strictly positive (or negative), then the two childrens’ bounds will match the parent’s and do not need to be recomputed.

### Zonotope Contraction

The accuracy of the zonotope prefilters is important, as large overapproximation error will lead to the computed overapproximation range of $x_n$ in zonotope *z* always overlapping zero, and thus performance similar to the Sim method. This effect is observed near the top of the curves in Fig. 2.

In order to improve accuracy, we propose a zonotope domain contraction approach, where the size of the zonotope set *z* is reduced while still maintaining an overapproximation of the exact star set $\theta$. As discussed before, computing exact intersections of zonotopes is generally impossible when splitting (lines 28–29 in Algorithm 2). However, we can lower our expectations and instead consider other ways to reduce the size of zonotope *z* while maintaining $\theta \subseteq z$.

To do this, we use a slightly different definition of a zonotope, which we refer to as an *offset zonotope*. Instead of an affine transformation of the $[-1, 1]^p$ box, an offset zonotope is an affine transformation of an arbitrary box, $[l_1, u_1] \times \ldots \times [l_p, u_p]$, where each upper bound $u_i$ is greater than or equal to the lower bound $l_i$. As this corresponds to an affine transformation of the $[-1, 1]^p$ box, offset zonotopes are equally expressive as ordinary zonotopes. Optimization over offset zonotopes can also be done using a simple summation, but instead of using Eq. 2, we use the following modified equation:

5 $$\begin{aligned} x_i^* = {\left\{ \begin{array}{ll} u_i v_i, &{} \text {if } u_i v_i \cdot g_i \ge l_i v_i \cdot g_i \\ l_i v_i, &{} \text {otherwise} \end{array}\right. } \end{aligned}$$

Using offset zonotopes allows for some memory savings in the algorithm. The initial zonotope can be created using a zero vector as the zonotope center and the identity matrix as the generator matrix, the same as the initial input star set. In fact, with this approach, since the affine transformations being applied to the zonotope *z* and star set $\theta$ are identical, the centers and generator matrices will always remain the same, so that we only need to store one copy of these.

Beyond memory savings, with offset zonotopes we can consider ways to reduce the zonotope’s overapproximation error when adding constraints to $\theta$. The proposed computations are done after splitting (lines 28–29 in Algorithm 2), each time an extra constraint gets added to the star set’s polytope *P*. The new linear constraint in the output space ($x_n \le 0$ or $x_n \ge 0$) is transformed to a linear constraint in the initial space using Eq. 4. We then try to contract the size of the zonotope’s box domain by increasing each $l_i$ and reducing each $u_i$, while still maintaining an overapproximation of the intersection. We consider two ways to do this which we call Contract-LP and Contract-Simple.

In Contract-LP, linear programming is used to adjust each $l_i$ and $u_i$. Since the affine transformations for the star set $\theta$ and the zonotope *z* are the same, *z* is an overapproximation if and only if the star set’s polytope *P* is a subset of *z*’s initial domain box $[l_1, u_1] \times \ldots \times [l_p, u_p]$. Thus, we can compute tight box bounds on *P* using linear programming, and using this box as the offset zonotope’s initial domain box. This will be the smallest box that is possible for the current affine transformation while still maintaining an overapproximation. This approach, however, requires solving 2*p* linear programs, which may be expensive.

Another approach is possible without invoking LP, which we call Contract-Simple. Contract-Simple overapproximates the intersection by considering only the new linear constraint. This is a problem of finding the smallest box that contains the intersection of an initial box and a single halfspace, which can be solved geometrically without LP solving (see Appendix A for an algorithm).

**Fig. 5.**
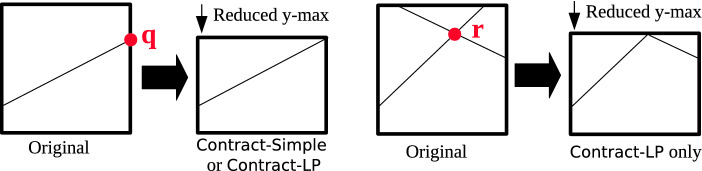
Both Contract-Simple and Contract-LP can find point *q* to contract a zonotope’s initial box (left), but only Contract-LP can find point *r* (right), as it requires reasoning with multiple linear constraints.

Since Contract-Simple only considers a single constraint, it can be less accurate than Contract-LP. An illustration of the two methods is given in Fig. 5, where the initial domain is a two-dimensional box. The thin lines are the linear constraints that were added to $\theta$, where all points below these lines are in the corresponding halfspaces. On the left, both Contract-Simple and Contract-LP can reduce the upper bound in the *y* direction by finding the point *q*, which lies at the intersection of one side of the original box domain and the new linear constraint. On the right, two constraints were added to the star $\theta$ (after two split operations), and they both must be considered at the same time to find point *r* to be able to reduce the upper bound in the *y* direction. In this case, only Contract-LP will succeed, as Contract-Simple works with only a single linear constraint at a time, and intersecting the original box with each of the constraints individually does not change its size.

Comparing the performance of the methods in Fig. 4, we see that the less-accurate but faster Contract-Simple works best for the ACAS Xu benchmarks. We expect both methods to take longer when the input set has more dimensions, but especially Contract-LP since it requires solving two LPs for every dimension.

**Correctness Justification:** The domain contraction procedures reduces the size of zonotope *z* while maintaining an overapproximation of the star set $\theta$. This can be seen since the affine transformations in *z* and $\theta$ are always the same, and every point in the star set’s initial input polytope *P* is also a point in the initial box domain of *z*. Since an overapproximation of $\theta$ is maintained, it is still sound to use *z* when determining the possible signs of a neuron’s output.

## Evaluation with Other Tools

We next compare the optimized implementation with other neural network verification tools. Our optimizations are part of the exact analysis mode of the nnenum tool available at https://github.com/stanleybak/nnenum. The artifact evaluation package for our measurements here is online at http://stanleybak.com/papers/bak2020cav_repeatability.zip.

We evaluate with the fully optimized method, using DFS local search, Zono-Sim prefilter, Eager bounds, Contract-Simple zonotope domain contraction. Further, we use a parallelized version of the algorithm, where the details of the parallalization are provided in Appendix B. With a 12-thread implementation (one for each core on our evaluation system), the algorithm can now verify all 180 ACAS Xu benchmarks from properties 1–4 within the 10 min timeout. All measurements are done on our Laptop system, with hardware as described in the first paragraph of Sect. 3. The complete measurement data summarized here is available in Appendix D.

**Fig. 6.**
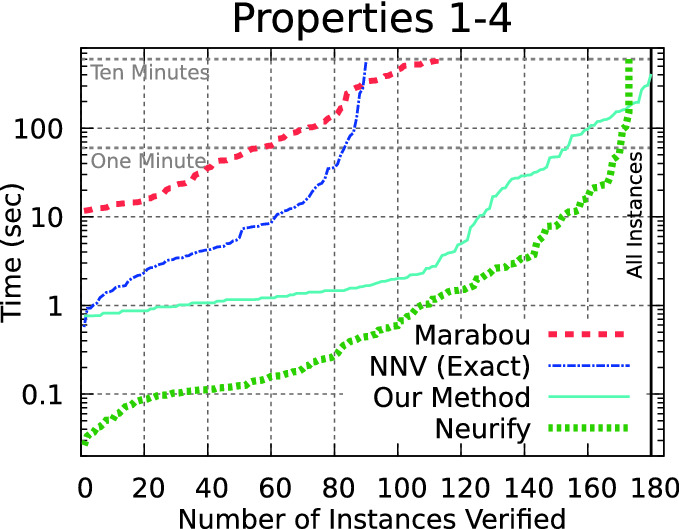
Our method verifies all the benchmarks, although Neurify is usually faster when it completes.

**Fig. 7.**
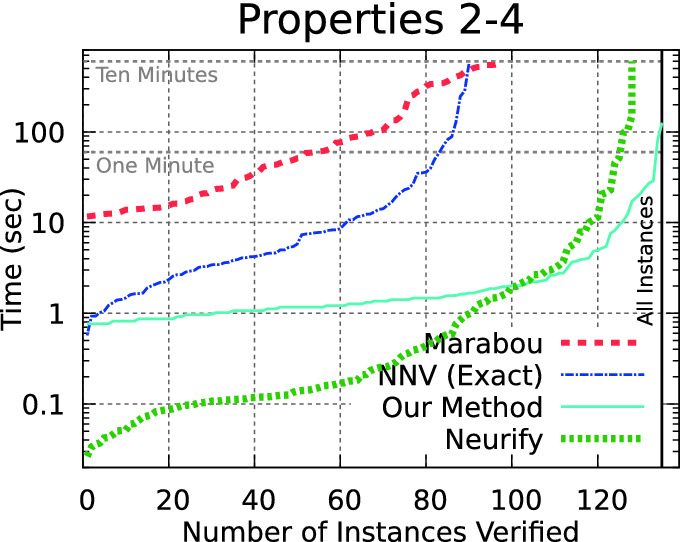
Without property 1, our approach is generally fastest when the runtime exceeds two seconds.

**ACAS Xu Properties 1–4.** We compare our method with Marabou [14] Neurify [26], and NNV [25]. Marabou is the newer, faster version of the Reluplex algorithm [13], where a Simplex-based LP solver is modified with special ReLU pivots^2^. Neurify is the newer, 20x faster version of the ReluVal algorithm [27], which does interval-based overapproximation, and splits intervals based on gradient information, ensuring the overapproximation error cannot cause to an incorrect result. NNV is the original Matlab implementation of the path enumeration method with star sets, available online at https://github.com/verivital/nnv. The verification result is consistent between the methods, which is a good sanity check for implementation correctness.

The comparison on ACAS Xu benchmarks on properties 1–4 is shown in Fig. 6. Our method is the only approach able to analyze all 180 benchmarks in less than 10 min, and outperforms both Marabou and NNV.

**Table 1.** Tool runtime (secs) for ACAS Xu properties 5–10.

Property	Net	Result	Our method	ERAN	Neurify	NNV exact	Marabou
5	1-1	SAFE	13	–	12	671	1969
6.1	1-1	SAFE	67	–	3	6230	12425
6.2	1-1	SAFE	76	–	1	7612	17755
7	1-9	UNSAFE	5948	–	804	–	–
8	2-9	UNSAFE	.7	–	64	–	–
9	3-3	SAFE	88	318	393	12576	15235
10	4-5	SAFE	12	–	1	457	2795

The comparison with Neurify is more complicated. In Fig. 6, Neurify was faster (when it finished) on all but the largest instances. One advantage of Neurify compared with the other tools is that if the unsafe set is very far away from the possible outputs of a neural network, it can prove safety quickly with a very coarse overapproximation. Path enumeration methods, on the other hand, explore all paths regardless of the distance to the unsafe set. This is especially relevant for ACAS Xu property 1, where the system is unsafe if the first output, clear-of-conflict, is greater than 1500 whereas, for example on network 1-1, this output is always smaller than 1. The meaning of this property is also strange: the absolute value of a specific output is irrelevant, as relative values are used to select the current advisory. Neurify is admittedly the clear winner for all the networks with this property.

When this property is excluded and instead only the more difficult properties 2–4 are considered (Fig. 7), a different trend emerges. Here, our method outperforms Neurify when analysis takes more than about two seconds, which we believe is an encouraging result. Further, part of the reason why Neurify can be very quick on the easier benchmarks (with runtime less than two seconds) is that our implementation incurs a startup delay of about 0.6 s simply to start the Python process and begin executing our script, by which time the C++-based Neurify can verify 80 benchmarks. We believe the more interesting cases are when the runtimes are large, and we outperform Neurify in these cases.

Finally, we compare with using single-set overapproximations for analysis. NNV provides an approximate-star method, where rather splitting, a single star set is used to overapproximate the result of ReLU operations. While fast when it succeeds, this strategy can only verify 68 of the 180 benchmarks. Furthermore, the benchmarks it verified were also quickly checked with exact path enumeration. Of the 68 verified benchmarks, the largest performance difference was property 3 with network 3-3, which took 3.1 s with exact enumeration and 1.2 s with single-set overapproximation. For these ACAS Xu benchmarks, overapproximation using a single set does not provide much benefit.

**Other ACAS Xu Properties.** Another recently proposed and well received analysis method is presented in the elegant framework of abstract interpretation using zonotopes, in tools such as AI$^2$ [7] or DeepZ [21]. These methods are single-set overapproximation methods, similar to the approximate-star method in NNV, but with strictly more error (see Fig. 2 in the NNV paper [24] and the associated discussion). As these methods have more error than approximate-star, and since approximate-star could only verify 68 of the 180 benchmarks, we do not expect these methods to work well on the ACAS Xu system.

However, a recent extension to these methods has been proposed where the overapproximation is augmented with MILP solving [22] to provide complete analysis. This has been implemented in the ERAN tool, publicly available at https://github.com/eth-sri/eran. According to current version of the README, ERAN currently only supports property 9 of ACAS Xu, so we were unable to try this method on the other ACAS Xu networks or properties. Verifying property 9 uses a hard-coded custom strategy of first partitioning the input space into 6300 regions and analyzing these individually. This problem-specific parameter presents a problem for fair timing comparison, as the time needed to find the splitting parameter value of 6300 is unknown and does not get measured.

Ignoring this issue, we ran a comparison on property 9 and network 3-3, the only network where the property applies. A runtime comparison for ERAN^3^ and the other tools is shown in Table 1. Surprisingly, our enumeration method significantly outperforms the overapproximation and refinement approaches both in Neurify and ERAN on this benchmark. Notice, however, that the original enumeration method in NNV is much slower than our method (about 150x slower in this case). Without the optimizations from this work, one would reach the opposite conclusion about which type of method works better for this benchmark. Both NNV and our method, however, report exploring the same number of paths, 338600 on this system.

For completeness, Table 1 also includes the other original ACAS Xu properties, which were each defined over a single network^4^. Both our method and Neurify completed all the benchmarks, although neither was best in all cases. Property 7 is particularly interesting, since the input set is the entire input space, so the number of path is very large. Hundreds of millions of paths were explored before finding a case where the property was violated.

## Related Work

As the interest in neural networks has surged, so has research in their verification. We review some notable results here, although recent surveys may provide more a thorough overview [15, 28]. Verification approaches for NNs can broadly be characterized into geometric techniques, SMT methods, and MILP approaches.

Geometric approaches, like this work, propagate sets of states layer by layer. This can be done with polytopes [6, 29] using libraries like the multi-parametric toolbox (MPT) [10], although certain operations do not scale well, in particular, affine transformation. Other approaches use geometric methods to bound the range of a neural network. These include $\text {AI}^2$ [7] and DeepZ [21] which propagate zonotopes through networks and are presented in the framework of abstract interpretation. ReluVal [27] and Neurify [26] also fall into this category, using interval symbolic methods to create overapproximations, followed by a refinement strategy based on symbolic gradient information. Some of these implementations are also sound with respect to floating-point rounding errors, which we have not considered here, mostly for lack of an LP solver that is both fast and does outward rounding. Other NN verification tools such as Reluplex, Marabou, ERAN, and NNV also use numeric LP solving. Another performance difference is that we used the free GLPK library for LP solving and some other tools used the commercial Gurobi optimizer, which is likely faster. Other refinement approaches partition the input space to detect adversarial examples [11], compute maximum sensitivity for verification [30], or perform refinement based on optimization shadow prices [20].

Mixed integer-linear programming (MILP) solvers can be used to exactly encode the reachable set of states through a ReLU network using the big-M trick to encode the possible branches [16, 23]. This introduces a new boolean variables for each neuron, which may limit scalability. The MILP approach has also been combined with a local search [5] that uses gradient information to speed up the search process.

SMT approaches include the Reluplex [13] and Marabou [14], which modify the Simplex linear programming algorithm by splitting nodes into two, which are linked by the semantics of a ReLU. The search process is modified with updates that fix the ReLU semantics for the node pairs. Another tool, Planet, combines the MILP approach with SAT solving and linear overapproximation [6].

Here, we focused on input/output properties of the neural network, given as linear constraints. This formulation can check for adversarial examples [9] in image classification within some $L_\infty$ norm of a base image, which are essentially box input sets. Other more meaningful semantic image perturbations such as rotations, color shifting, and lighting adjustments can also be converted into input/output set verification problems [19].

## Conclusions

One of the major successes of formal verification is the development of fast model checking algorithms. When talking about how improvements to model checking algorithms came about, Ken McMillan noted:

“Engineering matters: you can’t properly evaluate a technique without an efficient implementation.” [18]

With this in mind, we have strived to improve the practical efficiency of the complete path-enumeration method for neural network verification. Although the geometric path-enumeration method has been proposed before, we have shown that, by a sequence of optimizations, the method’s scalability can be improved by orders of magnitude.

One limitation is that we have focused on the ACAS Xu benchmarks. Although there is a risk of overfitting our optimizations to the benchmarks being considered, we believe these benchmarks are fairly general in that they contain a mix of safe and unsafe instances, where the original verification times varied from seconds to days. In particular, we believe these networks are similar to others being used in control tasks, in terms of number of inputs and network size. Further, practical considerations prevent us from considering too many more benchmarks; our measurements already need over five days to run.

Unreported here, we were also able to run the implementation on larger perception networks to analyze $L_\infty$ perturbation properties, networks with thousands of neurons and hundreds of inputs, which succeeds when the perturbation is sufficiently small. However, we believe path enumeration is the wrong approach for those systems, as the number of paths quickly becomes too large to enumerate. Instead, overapproximation and refinement methods would likely work best, and evaluating optimizations for these methods may be done in future work. One interpretation of the results presented here is that overapproximation and refinement methods still have significant room for improvement, as it is sometimes faster to explicitly enumerate benchmarks with millions of paths.

Many of the tools we have compared against also support more complicated network structures, with different layer types and nonlinear activation functions, whereas we only focused on the subclass of networks with ReLUs and fully-connected layers. We believe that this is an important enough subclass of neural networks that the results are still meaningful. Once the neural network verification community is more mature, we expect a standard input format and a set of categorized benchmarks will arise, similar to what has happened in the SMT [2], software verification [3], and hybrid systems [1] communities.

## Figures and Tables

**Fig. 8.**
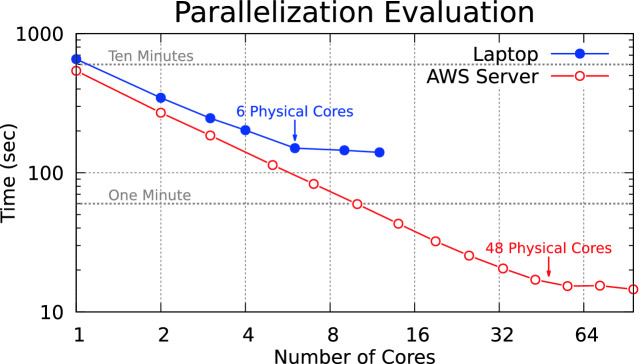
Doubling the number of cores roughly halves the computation time, up to the physical core count on each platform.

## A Box Bounds Algorithm for Box-Halfspace Intersection

The problem of computing the box bounds of an intersection of an initial box and a single halfspace can be computed without LP. Consider a *p*-dimensional initial box defined with lower and upper bounds $[l_1, u_1] \times \ldots \times [l_p, u_p]$. Call the constraint defining the halfspace $f \alpha \le \texttt {g}$, where $\alpha$ is a *p*-dimensional vector of variables, *f* is a *p*-dimensional vector with entries $f_1, \ldots , f_p$, and $\texttt {g}$ is a scalar.

Based on the signs of the signs of $f_1, \ldots , f_p$, we first find the vertex $v^*$ in the box that minimizes the dot product $f \cdot v^*$. This can be done by choosing the *i*th element of $v^*$ as:

6 $$\begin{aligned} v_i^* = {\left\{ \begin{array}{ll} l_i, &{} \text {if } f_i \ge 0 \\ u_i, &{} \text {otherwise} \end{array}\right. } \end{aligned}$$

If $f \cdot v^* > \texttt {g}$, then the intersection is the empty set. Otherwise, we attempt to contract in each of the *p* dimensions one-by-one.

For dimension *i*, if the lower bound was used to define $v_i^*$, then we attempt to decrease $u_i$. If the upper bound was used to define $v_i^*$, then we attempt to increase $l_i$. This is done by finding the point on the edge of the box which intersects the halfspace (point *q* in Fig. 5). Without loss of generality, assume the lower bound of dimension *i* defined $v_i^*$. The intersection point *q* is given by $(v_1^*, v_2^*, \ldots x, \ldots v_p^*)$, where value of the *i*th coordinate, *x*, can be determined from the single-variable equation $q \cdot f = \texttt {g}$. If $f_i$ was zero, then this equation has no solution, and we cannot contract in this dimension (the half-space and the box edge where *q* must lie do not intersect). Otherwise, if we solve for *x* and find $x < u_i$, then we reduce $u_i$, setting it to *x*. The process repeats for every other dimension.

## B Parallelization

The proposed approach can be parallelized in many ways. Here, we propose and evaluate a work-stealing strategy, where each thread maintains a local set of computation-state tuples and runs the high-level algorithm. Periodically, the number of tuples in each local set are communicated using a shared data structure, and if some worker thread has no work remaining, the other threads will push some of their local computation-state tuples to a shared global queue.

For this evaluation, we used the usual system setup described in the first paragraph of Sect. 3, which we label Laptop. In addition, to see the effect of more cores, we rented a c5.metal EC2 instance from Amazon Web Services, which we refer to as AWS Server. This setup ran Ubuntu 18.08, and included a dual Intel(R) Xeon(R) Platinum 8275CL processor running at 3.0 GHz, with a total of 48 physical cores (96 with hyperthreading) and 384 GB of main memory.

To evaluate parallelism, we needed to use a benchmark with sufficient difficulty where computation time dominates. For this, we chose ACAS Xu network 4-2 with specification 2. In an earlier ACAS Xu evaluation [14], this property timed out (>55 min) or ran out of memory for every tool analyzed. The single-threaded runtime on the Laptop platform with our enumeration approach was 655 s (about 11 min), which enumerated 484555 paths in the network.

An evaluation where we adjusted the number of cores available to the computation process for each of the two platforms is shown in Fig. 8. The AWS Server platform was faster than the Laptop setup and, with all the cores being used, could enumerate the same 484555 paths in about 15 s. The linear trend on the log-log graph shows continuous improvement as more cores are added, up to the physical-core limit on each platform. The gains from hyperthreading are comparatively smaller. Even using all the cores, about 90% of the computation time was in the step function, as opposed to managing shared state. With more cores, further improvement through additional parallelization is likely possible.

**Correctness Justification:** Parallelization explores the same set of states, just in a different order.

## C Full Optimization Data

See Table 2.

**Table 2.** Runtimes (sec) for each optimization. Dashes (  ) are timeouts (10 min).

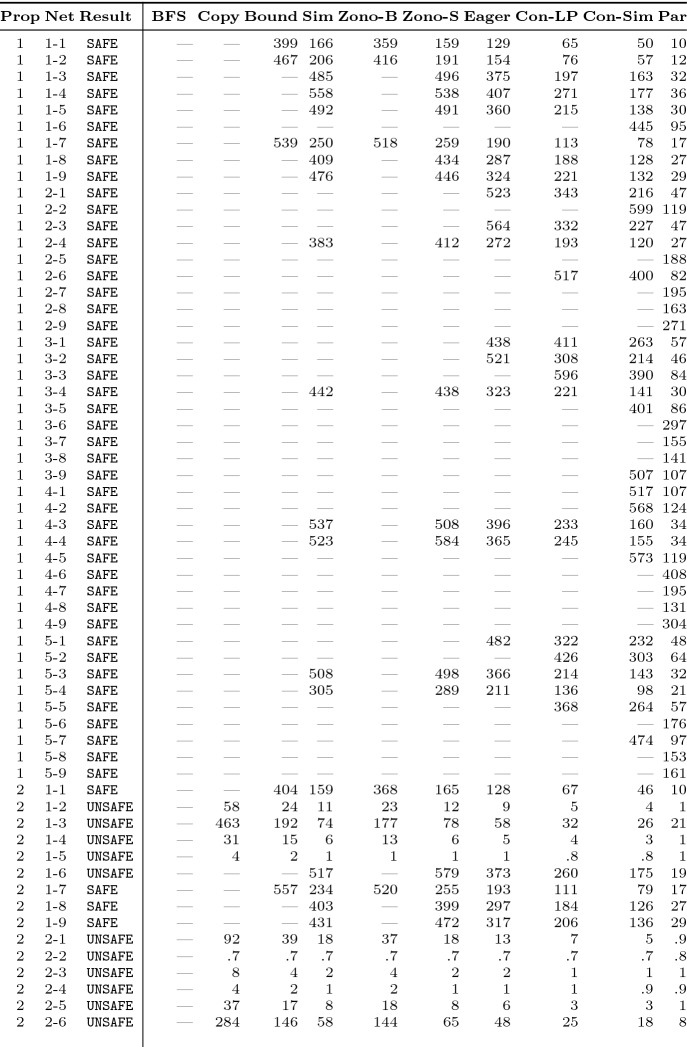

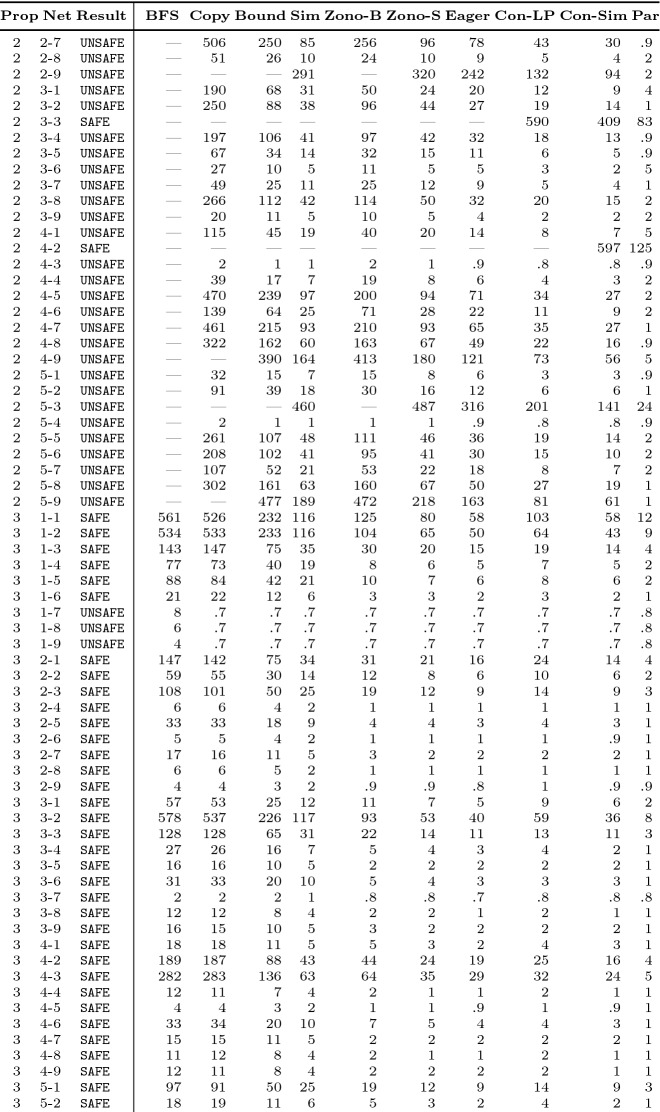

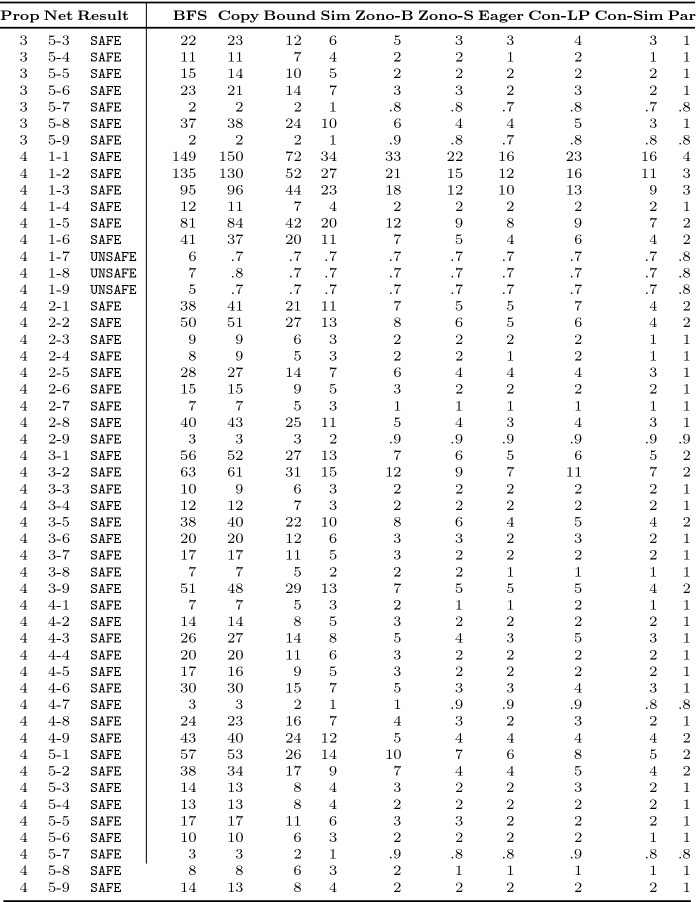

## D Full Tool Comparison Data

This section contains the complete data measured in the optimization improvements from Sect. 3 (Table 3).

**Table 3.** Runtimes (sec) for each tool. Dashes (  ) are timeouts (10 min).

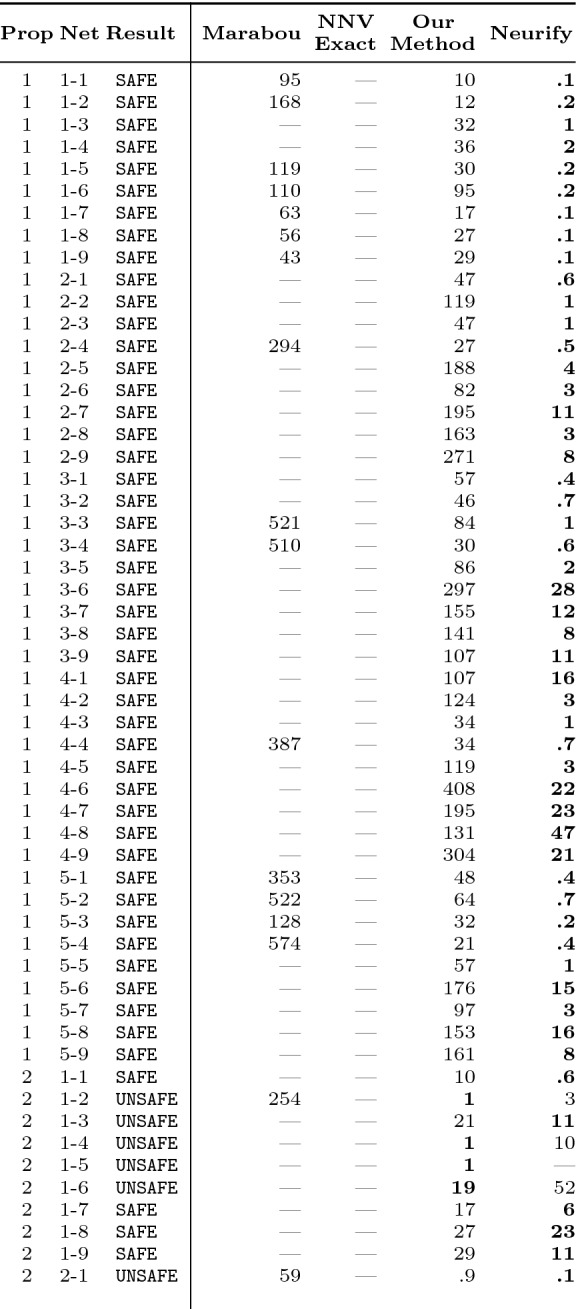

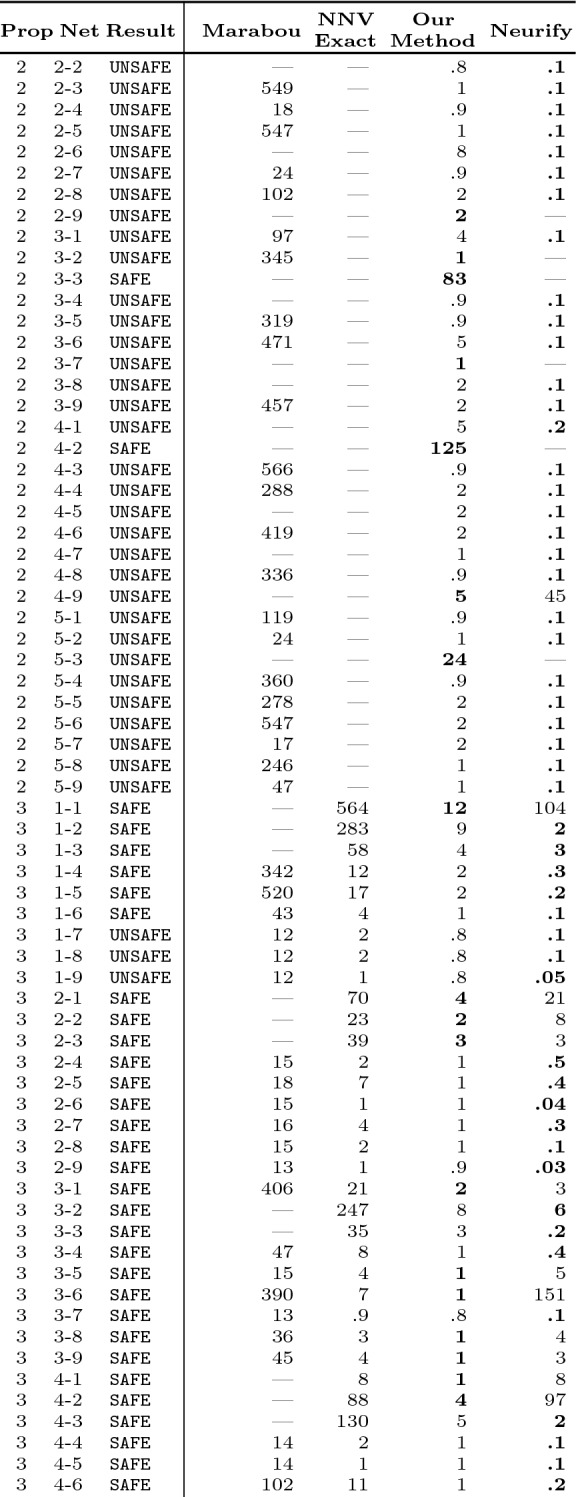

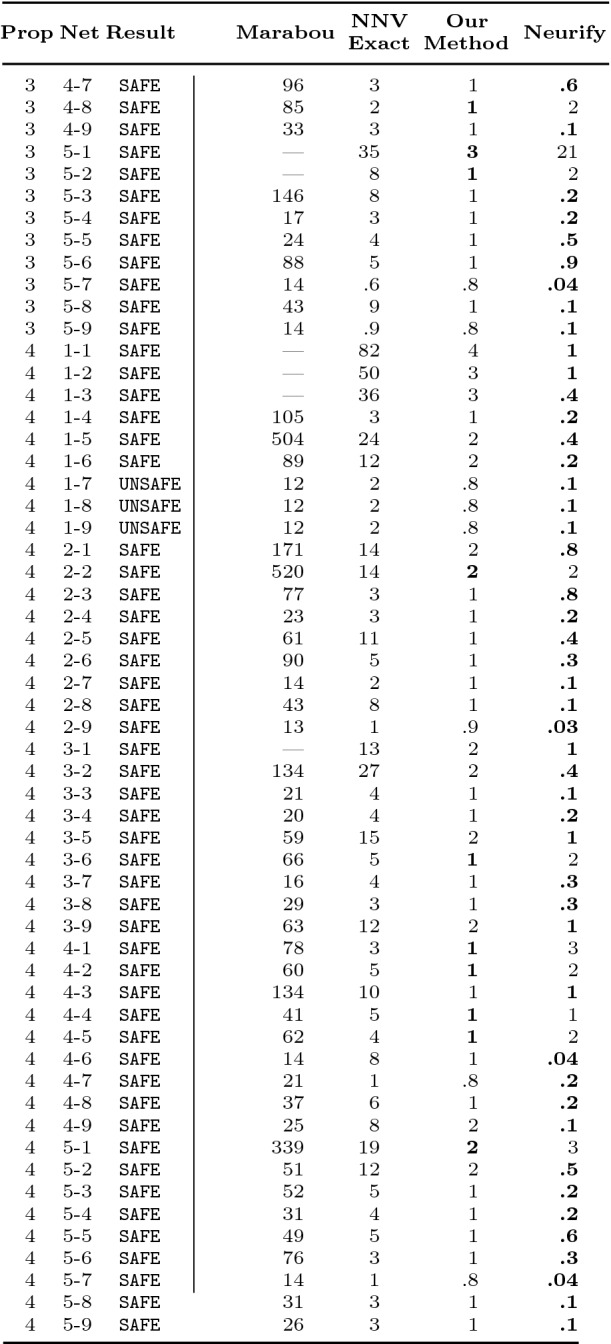

## Footnotes

1 The DFS method solves every benchmark that can be solved with BFS. Appendix C contains the complete results.

2 For Marabou, we used the faster parallel divide-and-conquer mode with arguments as suggested in the paper [14]:  .

3 For ACAS Xu analysis, we used the following arguments provided by the ERAN authors:  .

4 Property 6’s input set was a disjunction of two boxes which we split into two cases.
